# Experimental Investigation on the Development of Environmentally Friendly Chitosan Quaternary Shale Inhibitor

**DOI:** 10.3390/polym18050561

**Published:** 2026-02-26

**Authors:** Zhifeng Duan, Yong Ouyang, Daichun Si, Zhanying Huang, Yu Zhou, Cheng Hui

**Affiliations:** 1 National Engineering Laboratory for Exploration and Development for Low-Permeability Oil & Gas Fields, Xi’an 710018, China; 2 Oil and Gas Technology Research Institute, PetroChina Changqing Oilfield Company, Xi’an 710018, China

**Keywords:** chitosan, shale inhibitor, quaternary ammonium salt, environmental protection, water-based drilling fluid

## Abstract

With the increasingly stringent environmental regulations, the development of high-performance and eco-friendly shale inhibitors for water-sensitive formations has become an urgent priority. Chitosan, a renewable biopolymer derived from chitin, has inherent potential as a shale inhibitor but is limited by low water solubility and suboptimal inhibition efficiency. To overcome these limitations, cationic quaternary ammonium groups were grafted onto chitosan through etherification with 3-chloro-2-hydroxypropyltrimethylammonium chloride (CHA), yielding chitosan quaternary ammonium chloride (QASC). Systematic evaluation through linear swelling, rolling recovery, and bentonite inhibition tests revealed QASC’s superior performance. Notably, 1% QASC reduced bentonite swelling to 28.1% after 16 h, outperforming 5% KCl (48.2%) and 1% polyetheramine (41.1%). Remarkably, QASC achieved 88.4% shale recovery at 150 °C significantly exceeding the values for polyetheramine (52%) and pure water (13.2%). Mechanistic analysis revealed that QASC inhibits clay hydration through dual mechanisms: (1) electrostatic and hydrogen-bond mediated adsorption on clay surfaces, effectively neutralizing surface charges and diminishing hydration films; (2) intercalation into clay interlayers to create a physical barrier against water invasion. This synergistic combination ensures stable inhibitory performance under elevated temperatures. Given its enhanced biodegradability, QASC emerges as a sustainable alternative to conventional inhibitors, effectively addressing the dual challenges of technical performance and environmental compatibility in shale gas drilling operations.

## 1. Introduction

Shale oil and gas, as the core area of global unconventional energy development, primarily rely on the technical system dominated by “extended-reach horizontal wells + fracturing development.” However, since shale contains various types of clays and clay minerals, the direct use of water-based drilling fluids can lead to shale swelling, resulting in reduced strength and wellbore instability. Shale swelling is a serious issue that may, in some cases, disrupt the entire drilling process [1,2]. Due to the high water sensitivity of shale, oil-based or synthetic-based drilling fluids exhibit superior inhibition performance compared to water-based drilling fluids [3,4]. Nevertheless, their widespread application is limited by high costs and environmental hazards.

The addition of shale inhibitors to water-based drilling fluids is a critical technical approach for effectively suppressing shale hydration and swelling. Initially, inorganic salt inhibitors such as sodium chloride, potassium chloride, and calcium chloride were employed [5]. These inhibitors function by compressing the thickness of the diffuse double layer on shale surfaces, thereby stabilizing the shale. However, frequent use of high-concentration potassium chloride in water-based drilling fluids can lead to loss of control over the system’s rheological and filtration properties [6,7]. Given that amine cations exhibit inhibition characteristics similar to potassium ions, this has spurred the development of polyamine-based shale inhibitors [8,9,10,11]. Common polyamine inhibitors include polymers such as polyacrylamide (PAM) and partially hydrolyzed polyacrylamide (HPAM). Polyamine inhibitors are widely utilized due to their superior inhibition performance, as they neutralize the negative charges on clay surfaces and promote bridging adsorption between clay platelets to stabilize shale [12,13,14]. However, polyamine inhibitors are relatively costly and tend to degrade at high temperatures (>150 °C), resulting in diminished inhibition efficacy. Nanoparticles are also commonly used as shale inhibitors in field applications. They adhere to shale surfaces and form a barrier, inhibiting water molecule penetration into the shale [15,16]. Nevertheless, nanoparticles are prone to agglomeration and require dispersants to maintain stability.

In recent years, environmental regulations and laws have become increasingly stringent, requiring that chemicals used in drilling operations within the oil and gas industry must have minimal ecological impact. Consequently, higher demands have been placed on drilling fluid additives [17]. As a result, many researchers have focused on developing highly efficient and environmentally friendly inhibitors for water-based drilling fluids to mitigate shale hydration issues [18].

The development of high-performance, non-toxic, biodegradable, and cost-effective shale inhibitors is crucial. Natural material extracts appear to be promising alternatives for producing low-cost inhibitors [19,20,21]. Chitosan, a natural polymeric material derived from the deacetylation of chitin, is a positively charged natural polysaccharide containing reactive groups such as hydroxyl and amino groups in its structure [22]. Due to its excellent biodegradability and environmental friendliness, it was explored early for applications in drilling fluids. Studies have found that the amino and hydroxyl groups on its molecular chain can adsorb onto clay surfaces through hydrogen bonding, inhibiting shale hydration and swelling. The structural characteristics of chitosan meet the requirements for high-performance shale inhibitors [23]. However, due to excessively strong intramolecular hydrogen bonding, water molecules struggle to disrupt chitosan’s molecular structure, resulting in poor water solubility that significantly limits its applications [24]. Cationic modification of chitosan through etherification reactions is one of the most effective approaches to enhance both its water solubility and inhibition performance [25].

In this study, a quaternary ammonium salt of chitosan (QASC) was synthesized as an environmentally friendly shale inhibitor for water-based drilling fluids. This was achieved by grafting quaternary ammonium groups onto chitosan using the cationic etherifying agent 3-chloro-2-hydroxypropyltrimethylammonium chloride to enhance its inhibitory properties and water solubility. Furthermore, interactions between bentonite and QASC were investigated through multiple experimental methods, elucidating the mechanism by which QASC inhibits bentonite swelling and shale dispersion.

## 2. Materials and Methods

### 2.1. Experimental Materials

Oligochitosan (Degree of Deacetylation ≥95%), 3-chloro-2-hydroxypropyltrimethylammonium chloride (CHA, purity ≥65%), isopropyl alcohol (purity ≥98%), and polyetheramine (Mn = 230) were purchased from Shanghai Macklin Biochemical Co., Ltd., Shanghai, China. Sodium bentonite (industrial grade) was supplied by Weifang Huawei Bentonite Co., Ltd., Weifang, China. Shale was collected from Songlin County, China. Other common experimental chemicals were also obtained from Shanghai Macklin Biochemical Technology Co., Ltd., Shanghai, China.

### 2.2. Experimental Apparatus

CPZ-II Dual-Cell Shale LK Linear Swell Meter (Qingdao Jiaonan Analytical Instrument Factory, Qingdao, China); Six-Speed Rotational Viscometer (Qingdao Haidatong Specialized Instrument Co., Ltd., Qingdao, China); Mastersizer 3000 Laser Particle Size Analyzer (Malvern Panalytical Ltd., Worcestershire, UK); Zetasizer Nano Z90 Nanoparticle Size and Zeta Potential Analyzer (Malvern Panalytical Ltd., Worcestershire, UK); FlashSmart Elemental Analyzer (Thermo Fisher Scientific Inc., Waltham, MA, USA); TGA-2 Thermogravimetric Analyzer (Mettler Toledo Instrument Co., Greifensee, Switzerland).

### 2.3. Experimental Methods

#### 2.3.1. Preparation of QASC Inhibitor

**Figure 1:**
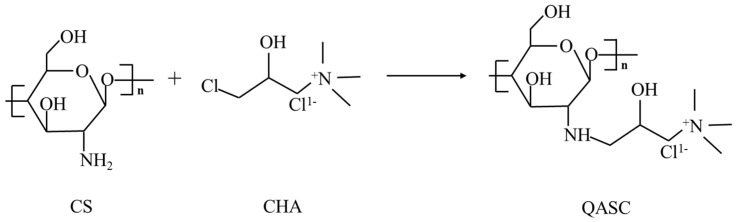
Schematic diagram of QASC.

A mixture of 100 mL isopropyl alcohol and 10 mL aqueous sodium hydroxide solution (40% *w*/*w*) was added into a three-necked flask. Under continuous stirring, 2 g chitosan was introduced. The system was then heated to 60 °C. After reacting for 2 h, the mixture was heated to 70 °C. Then, 8 mL of a solution of 3-chloro-2-hydroxypropyltrimethylammonium chloride (CHA) was added dropwise using a dropping funnel. The mixture was condensed under reflux for 10 h. Subsequently, the resulting solution was washed sequentially with ethanol and acetone (3 × 50 mL each) to remove impurities, followed by vacuum filtration. Finally, the obtained product was freeze-dried for 12 h. The dried product was ground into a fine powder to yield QASC in powder form. The reaction pathway is illustrated in Figure 1.

#### 2.3.2. Elemental Analysis

The elemental compositions of carbon (C) and nitrogen (N) in the synthesized QASC were quantitatively determined and comparatively analyzed against pristine chitosan using a FlashSmart elemental analyzer (Thermo Fisher Scientific, Waltham, MA, USA, combustion temperature: 950 °C, detection limit: 0.01 wt%) to assess the degree of quaternary ammonium group substitution.

#### 2.3.3. FTIR Analysis

FTIR analysis was performed to characterize the structure of QASC, with a scanning range of 4000–400 cm^−1^ and a resolution of 4 cm^−1^. The characteristic absorption peaks were analyzed to confirm the chemical structural stability.

#### 2.3.4. Thermogravimetric Analysis

The thermal degradation behavior of QASC was quantitatively characterized by simultaneous thermogravimetric analysis (STA) using a Netzsch TG 209 F1 Libra system (Selb, Germany) equipped with platinum crucibles. Approximately 5 mg of sample was heated from 40 to 700 °C under high-purity N_2_ (99.999%, flow rate: 50 mL·min^−1^) at a programmed heating rate of 10 °C min^−1^ with ±0.1 °C temperature resolution, monitoring mass loss profiles in real time.

#### 2.3.5. Solubility Test of QASC

CS and QASC (1 g each) were placed in separate beakers. Distilled water (100 mL) was gradually added to each beaker under constant stirring, and dissolution behavior was observed in real time at 25 °C.

#### 2.3.6. Linear Expansion Test

Sodium bentonite powder (10.0 ± 0.1 g) was homogeneously packed into a stainless steel die (Φ25 × 50 mm) and uniaxially compressed at 10 MPa with 5 min dwell time using a servo-hydraulic press (Instron 5869, Norwood, MA, USA, load cell accuracy: ±0.5% FS). The resultant cylindrical pellet was aged at 25 ± 1 °C/60% RH for 24 h prior to testing. The conditioned pellet was mounted on a dual-channel linear swell meter equipped with temperature-controlled test chambers. Aqueous inhibitor solutions (1 wt%) were injected into the chamber until complete immersion of the pellet. Real-time axial displacement was recorded at 1 Hz sampling frequency over 16 h isothermal period (25 ± 0.5 °C) using LabVIEW-based data acquisition system v.Q1 and the swelling height vs. time curve was plotted to quantify the hydration inhibition efficiency of every kind of solution.

#### 2.3.7. Shale Rolling Recovery Test

Shale cuttings (20 ± 0.05 g, 6–10 mesh) were loaded into 316 L stainless steel aging cells (OFITE Model 170-50, Houston, TX, USA). Aqueous inhibitor solution (350 mL, 1 wt%) was injected into the cell via pressure-compensated syringe, followed by helium purging (3 × 5 min) to eliminate atmospheric interference. The sealed cells were subjected to simulated downhole conditions in a roller oven at 120 ± 1 °C for 16 h, replicating triaxial stress states via formation pressure simulation module. After cooling to ambient temperature (25 ± 1 °C), the cuttings slurry was quantitatively transferred to a 40-mesh brass sieve and rinsed with deionized water (3 × 200 mL) under 0.2 MPa nitrogen pressure. Retained cuttings (>40 mesh) were dried in a convection oven until mass stabilization (<0.1% variation over 1 h intervals). Rolling recovery (RR) was calculated as Equation (1).

RR (%) = (m_retained_/m_initial_) × 100% (1)

where m_retained_ denotes post-test mass (g) and m_initial_ represents initial cuttings mass.

#### 2.3.8. Bentonite Slurry Inhibition Test

Aqueous inhibitor solutions (350 mL) with gradient concentrations (0.5, 1.0, 1.5, 2.0 wt%) were prepared using deionized water (ASTM Type I). Sodium bentonite was homogeneously dispersed into the solutions to achieve bentonite-to-fluid ratios of 4–20 wt% (step: 4%) via high-shear mixing. The formulated slurries were transferred into 316 L stainless steel aging cells and subjected to simulated downhole conditions in a roller oven (OFITE Model 304-00, Houston, TX, USA) at 150 ± 1 °C under axial rotation (20 ± 1 rpm) for 16 h. Post-aging slurries were sheared at 10,000 rpm (Silverson L5M-A, Silverston, Chesham, UK, 10 min) to ensure additive homogeneity, followed by equilibration at 25 ± 0.5 °C Plastic viscosity (PV) and yield point (YP) were determined using a Fann 35 viscometer, Houston, TX, USA (300 and 600 rpm readings) according to API Recommended Practice 13B-1. YP values were calculated via Equation (2):

(2) $$YP=\theta_{300}-\theta_{600}/2$$

#### 2.3.9. Preparation of Base Slurry

Sodium bentonite (40.00 ± 0.05 g) was gradually dispersed into 1000 mL deionized water under controlled shear over a 15 min period to prevent particle agglomeration. Anhydrous Na_2_CO_3_ (2.00 ± 0.01 g) was introduced into the suspension, followed by mechanical homogenization (30 min) to achieve complete ion-exchange. The activated slurry was transferred to nitrogen-purged containers and aged at 25 ± 1 °C for 24 h under quiescent conditions to facilitate montmorillonite lattice expansion, with periodic viscosity measurements confirming hydration completion (<5% variation over 2 h).

#### 2.3.10. Zeta Potential Measurement

Bentonite dispersions were prepared by incorporating gradient concentrations of QASC (0.1–2.0 wt%) into the base slurry. The mixtures were homogenized via high-shear mixing for 24 h to achieve adsorption equilibrium, followed by centrifugation to remove aggregates. Zeta potential measurements were conducted using a Zetasizer Nano ZS90 system, Malvern, UK.

#### 2.3.11. Particle Size Distribution Analysis

The volume-weighted particle size distribution of bentonite in QASC-containing suspensions (0.1–2.0 wt%) was determined using the Malvern Mastersizer 3000 laser particle size analyzer, Malvern, UK. Samples were prepared via identical protocol to zeta potential experiments.

#### 2.3.12. Compatibility Evaluation

A base slurry (350 mL) was prepared by sequential incorporation of treatment additives (Table 1) under continuous mechanical stirring (600 ± 50 rpm). Each additive was homogenized via high-shear mixing (10,000 rpm, 20 min) prior to subsequent additions. The formulated drilling fluid was subjected to thermal aging in a roller oven (150 ± 3 °C, 16 h), followed by cooling to ambient temperature and rehomogenized (10,000 rpm, 5 min). Rheological properties (pre-/post-aging) were characterized using a Fann-type rotational viscometer (Grace M3600, Grance Instrument Company, Houston, TX, USA) per API RP 13B-1, while fluid loss was quantified via an API filter press. The drilling fluid formulation is detailed in Table 1.

## 3. Results and Discussion

### 3.1. Structural Characterization of QASC

#### 3.1.1. Elemental Analysis Result

The degree of substitution (DS) of CHA on chitosan was quantitatively determined via CHNS elemental microanalysis. As summarized in Table 2, the unmodified chitosan exhibited 41.52 ± 0.15% carbon (C) and 7.80 ± 0.05% nitrogen (N), whereas the modified quaternary ammonium salt chitosan (QASC) showed decreased elemental contents of 36.42 ± 0.12% C and 7.16 ± 0.03% N. The DS value of 64.6 ± 1.2% was calculated according to Equation (3) based on the elemental analysis results, confirming successful N-quaternization.

(3) $$\frac{m{\left(C\right)}_{QASC}}{m{\left(N\right)}_{QASC}}=\frac{m{\left(C\right)}_{CS}+\frac{12n{\left(C\right)}_{CHA}}{M_{W\left(CHA\right)}}\times R}{m{\left(N\right)}_{CS}+\frac{14n{\left(N\right)}_{CHA}}{M_{W\left(CHA\right)}}\times R}$$

where R is defined as the degree of substitution of CHA on chitosan (CS). $m{\left(C\right)}_{QASC}$ and $m{\left(N\right)}_{QASC}$ represent the mass of carbon and nitrogen atoms per QASC molecule, respectively. $m{\left(C\right)}_{CS}$ and $m{\left(N\right)}_{CS}$ denote the mass of carbon and nitrogen atoms per chitosan molecule. $n{\left(C\right)}_{CHA}$ and $n{\left(N\right)}_{CHA}$ are the number of carbon and nitrogen atoms per CHA molecule. $M_{W\left(CHA\right)}$ is the molecular weight of CHA.

#### 3.1.2. Thermogravimetric Analysis Results

**Figure 2:**
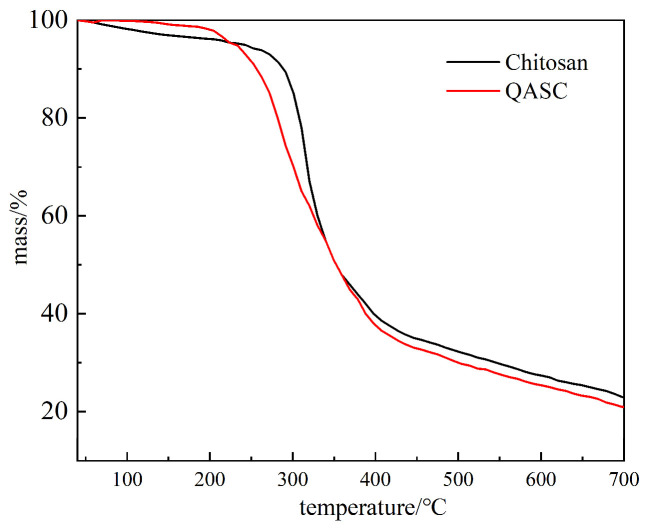
The thermogravimetric (TG) curves of chitosan and QASC.

The TGA results of chitosan (CS) and its cationic grafted derivative (QASC) are shown in Figure 2. Comparative analysis reveals that the thermal stability of QASC showed no significant alteration compared to unmodified chitosan after cationic group grafting. During the temperature ramp from 40 °C to 200 °C, both materials demonstrated minimal mass loss, attributable to the evaporation of surface-adsorbed water molecules. Notably, QASC maintained a consistently lower mass loss profile than unmodified chitosan until reaching 222 °C. Subsequent heating from 222 °C to 350 °C induced rapid decomposition in both specimens, with cumulative mass losses reaching 40.7% at 350 °C. Progressive heating to 700 °C resulted in accelerated degradation, culminating in final mass losses of 77.1% and 79.3% for CS and QASC, respectively, concurrent with substantial structural breakdown of the chitosan matrix. These findings confirm that QASC maintains exceptional thermal stability with negligible decomposition at temperatures below 200 °C.

#### 3.1.3. FTIR Analysis Results

**Figure 3:**
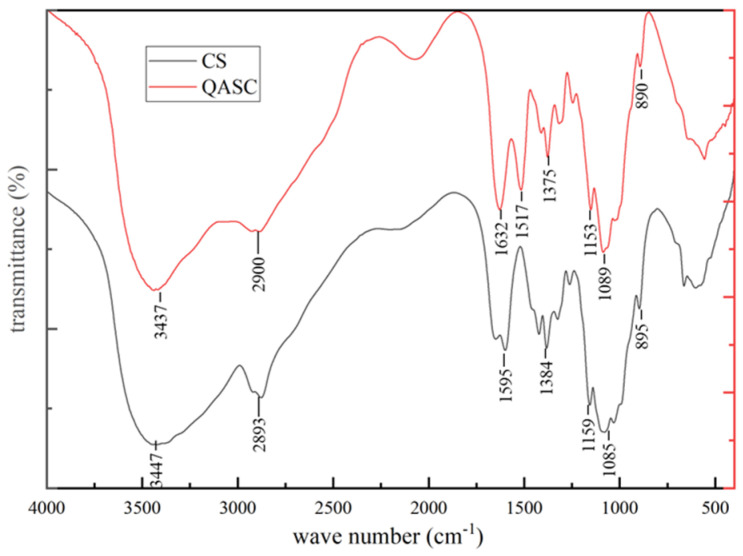
FTIR spectrum of CS and QASC.

As shown in Figure 3, in the FTIR spectrum of QASC, the emergence of a new characteristic peak at 1517 cm^−1^ is assigned to the asymmetric bending vibration of the methyl groups (-CH_3_) within the newly introduced quaternary ammonium groups (-N^+^(CH_3_)_3_). This assignment is corroborated by the significantly enhanced peak intensity observed in the ~2900 cm^−1^ region, which originates from the strengthened C-H stretching vibrations of the additional alkyl groups (predominantly -CH_3_) incorporated during the quaternization process. Concurrently, the characteristic absorption peak near 1595 cm^−1^ in native CS—attributed to the N-H bending vibration of the primary amino groups (-NH_2_)—exhibits complete disappearance in the QASC spectrum. Collectively, these spectroscopic changes demonstrate that the quaternization reaction primarily occurs at the amino sites on the chitosan molecular backbone.

#### 3.1.4. Solubility of QASC

**Figure 4:**
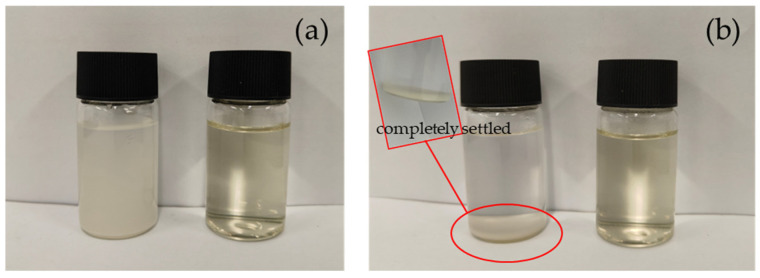
QASC water soluble figure ((**a**), well-stirred dispersion; (**b**), dispersion after 2 h of settling). (left) CS; (right) QASC).

As shown in Figure 4a, CS formed a heterogeneous suspension with immediate aggregation, while QASC yielded a homogeneous solution. As shown in Figure 4b, after 2 h quiescence, CS exhibited complete sedimentation with a clear supernatant, whereas QASC maintained full dissolution without precipitation.

### 3.2. Evaluation of Inhibitory Properties of QASC

#### 3.2.1. Results of the Shale Linear Expansion Experiment

**Figure 5:**
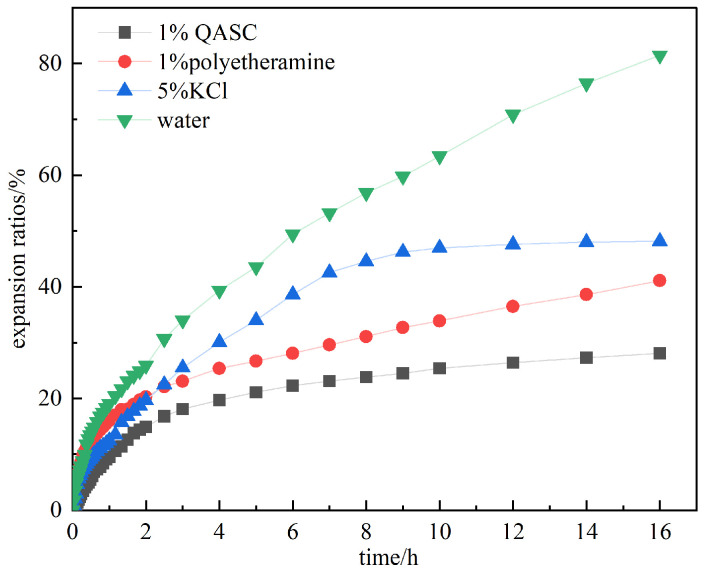
Swelling rate curves of bentonite in different inhibitor solutions.

The swelling kinetics of sodium bentonite tablets in various inhibitor solutions and deionized water are systematically shown in Figure 5. Pristine bentonite demonstrated characteristic hydration swelling behavior in aqueous environments. Initial water molecule penetration into the interlamellar spaces induced rapid hydration expansion, as evidenced by the steep upward trend in the expansion rate curve during the first 2 h. This swelling process exhibited progressive deceleration with time elapsed, reaching an equilibrium expansion ratio of 81.5% after 16 h of water immersion. Comparative analysis revealed that inhibitor-containing solutions significantly suppressed clay swelling, with measured linear expansion ratios of 48.2% (5% KCl), 41.1% (1% polyether amine), and 28.1% (1% QASC). Notably, QASC-modified systems showed 65.5% reduction in final expansion volume relative to control samples, demonstrating optimal inhibitor efficiency.

#### 3.2.2. Results of the Shale Rolling Recovery Experiment

**Figure 6:**
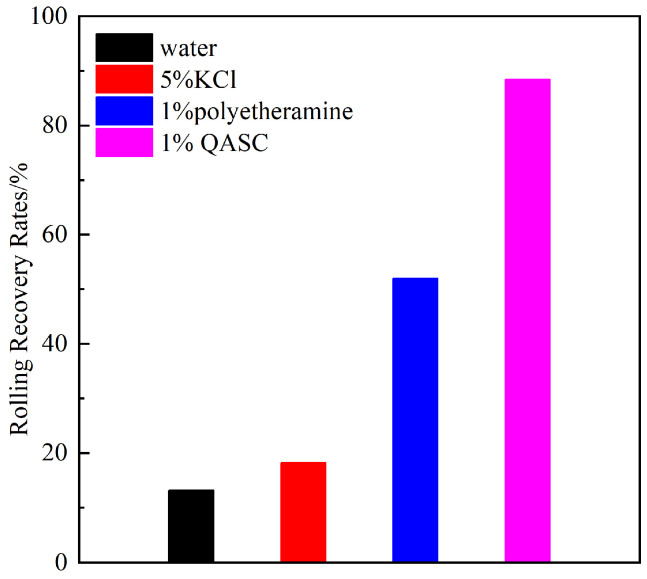
Shale rolling recovery rate in different inhibitor solutions.

The rolling recovery rates of cuttings in different solutions after thermal aging at 150 °C are illustrated in Figure 6. Following 16 h of high-temperature thermal aging, severe hydration and dispersion of cuttings resulted in a shale recovery rate of only 13.2% in deionized water. In the 5% KCl solution, the recovery rate slightly increased compared to pure water, indicating limited inhibitory efficacy of KCl against hydration and dispersion for this shale type. The recovery rate significantly improved to 52% in the 1% polyetheramine (PEA) solution. Notably, the 1% QASC solution achieved a markedly higher recovery rate of 88.4%, demonstrating its superior performance in suppressing shale hydration and dispersion. These recovery rate results align with the linear expansion experimental data, confirming that QASC exhibits exceptional inhibitory capabilities against both hydration dispersion and swelling of sodium bentonite.

#### 3.2.3. Results of the Inhibiting Bentonite Slurry Formation Experiment

**Figure 7:**
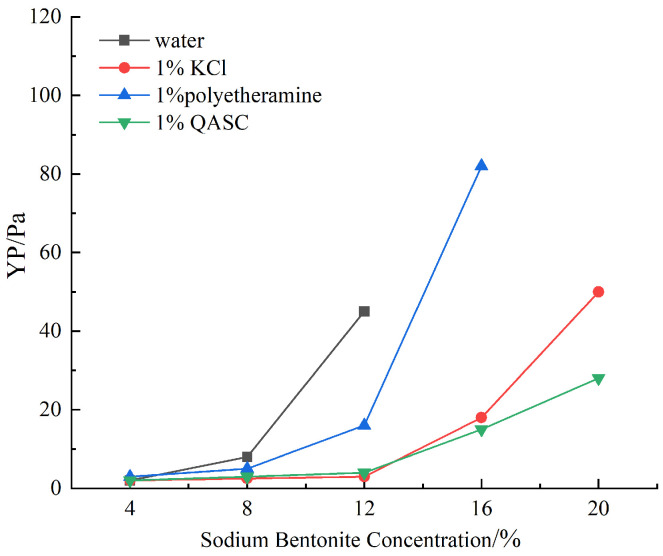
Variation of bentonite YP with concentration in different inhibitor solutions.

As shown in Figure 7, the inhibitory efficacy of shale stabilizers on bentonite hydration-dispersion processes was quantitatively assessed through standardized slurry inhibition testing. Figure 5 illustrates the variation in yield point (YP) of bentonite slurries with increasing bentonite content in deionized water and various inhibitor solutions. In pure water, the YP of a 12% sodium bentonite slurry became unmeasurably high after aging at 150 °C. In contrast, the YP values in inhibitor-containing solutions were significantly lower. When the bentonite content was below 12%, the YP differences among the inhibitor solutions were minimal. However, as the bentonite content increased further, marked YP variations emerged. At equivalent concentrations, the maximum bentonite content in polyetheramine (PEA) solution reached 16%, while both KCl and QASC solutions accommodated up to 20% bentonite. Notably, the YP of bentonite slurry in QASC solution was lower than that in KCl, indicating superior inhibitory performance of QASC. The swelling between bentonite crystallites is a primary factor influencing slurry performance. QASC, bearing positively charged functional groups, effectively neutralizes the surface negative charges of bentonite particles. This charge neutralization reduces interparticle electrostatic repulsion, promoting particle aggregation and flocculation, thereby suppressing hydration-induced expansion between bentonite particles.

### 3.3. Microstructural Analysis

#### 3.3.1. Results of Zeta Potential Measurement

**Figure 8:**
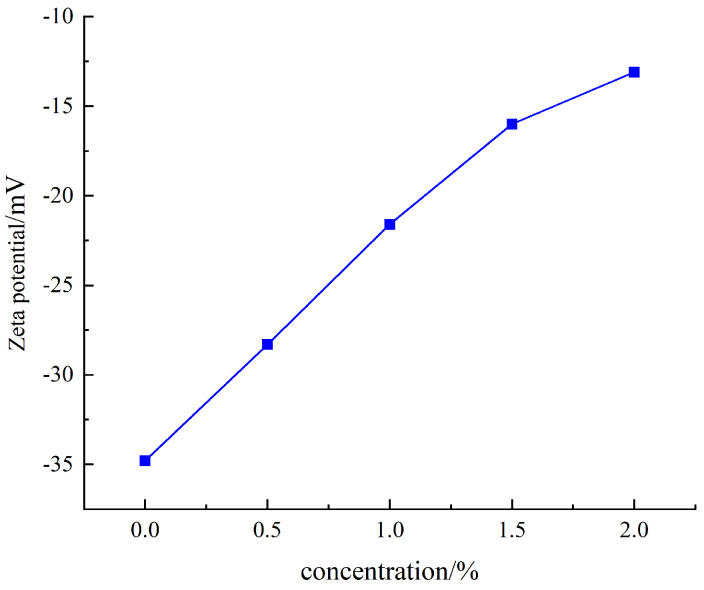
Zeta potential of sodium bentonite as a function of QASC concentration.

In aqueous environments, clay particles typically exhibit a negative surface charge due to isomorphous substitution in their crystal lattice, where higher-valent cations are replaced by lower-valent cations. The surface potential of clay directly governs its hydration, dispersion, swelling, and flocculation behaviors. Ionic group-containing inhibitors like QASC (quaternary ammonium salt compound) modulate the zeta potential, thereby altering the colloidal stability of clay particles. Generally, bentonite with a zeta potential absolute value exceeding 30 mV is considered to possess excellent dispersion stability. As shown in Figure 8, the zeta potential of sodium bentonite dispersion evolves with increasing QASC concentration. In pure water, sodium bentonite exhibits a zeta potential of −34.5 mV. Upon QASC addition, the absolute zeta potential progressively decreases, reaching −13.1 mV at 2 wt% QASC concentration. This demonstrates QASC’s effectiveness in reducing the zeta potential magnitude, consequently destabilizing the sodium bentonite colloid. The mechanism arises from the quaternary ammonium cationic groups in QASC neutralizing the negative charges on bentonite surfaces, compressing the diffuse double layer, and diminishing the hydration film thickness around bentonite particles, ultimately suppressing bentonite hydration.

#### 3.3.2. Results of Particle Size Distribution Analysis

**Figure 9:**
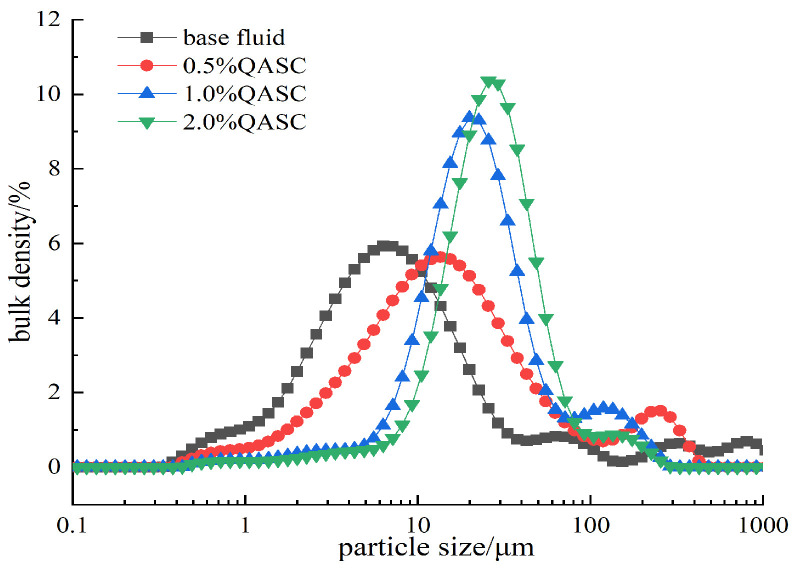
Effect of QASC concentration on the particle size distribution of bentonite.

As shown in Figure 9, the particle size distribution curve of bentonite gradually shifts to the right with increasing QASC concentration, indicating a progressive enlargement of bentonite particle size and effective suppression of its hydration and dispersion. According to zeta potential measurements, QASC inhibits bentonite dispersion by reducing the negative surface charge of clay particles and compressing the diffuse double layer [26,27]. Furthermore, when QASC is introduced into sodium bentonite dispersions, it intercalates into the interlayer spaces of bentonite particles, tightening the crystalline layers and promoting particle aggregation. This mechanism effectively mitigates the hydration-driven dispersion of sodium bentonite.

### 3.4. Analysis of the Inhibition Mechanism of QASC

**Figure 10:**
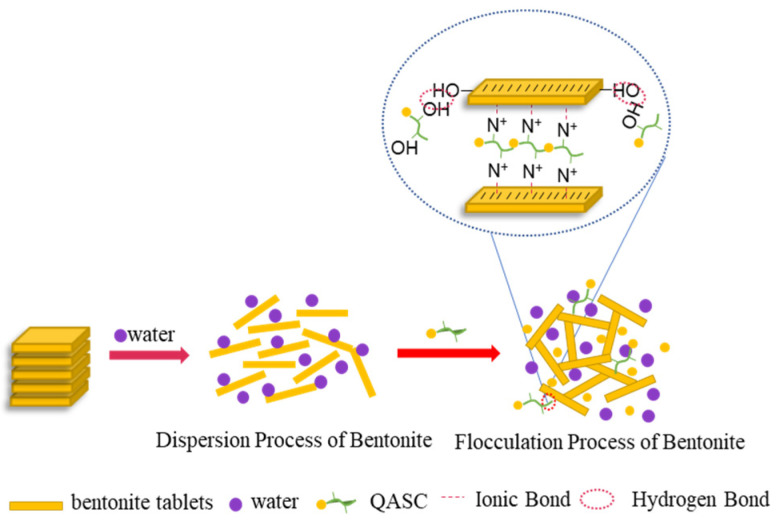
Mechanism diagram of QASC inhibiting bentonite hydration.

As shown in Figure 10, the QASC molecule possesses abundant active groups such as amino and hydroxyl groups, as well as quaternary ammonium cations. These structural features enable QASC to firmly adsorb onto clay particles via electrostatic interactions and hydrogen bonding. Upon adsorption, QASC effectively reduces the zeta potential of bentonite particles, compresses their diffuse double layer, and diminishes the thickness of the hydration film surrounding the clay. Furthermore, QASC intercalates into the interlayer spaces of clay crystals, where it tightly binds and contracts the crystalline layers. This intercalation physically blocks water penetration into the interlamellar regions, thereby suppressing both the hydration-induced dispersion of clay particles and the swelling of shale cuttings.

### 3.5. The Effects of QASC on Drilling Fluids

The rheological stabilization effects of QASC on the water-based drilling fluid system before and after thermal aging are quantitatively compared in Table 3. Pre-aging characterization revealed that the addition of QASC slightly increased the viscosity of the drilling fluid, marginally enhanced its shear force, and exhibited minimal impact on filtration performance. Post-aging analysis demonstrated that the influence of QASC on the drilling fluid remained similar to those observed before aging. These parameters confirm QASC’s excellent compatibility and thermal endurance in bentonite-based drilling fluids, achieving optimal equilibrium between rheological modification and filtration control.

## 4. Conclusions

1. A novel environmentally friendly inhibitor, quaternary ammonium salt of chitosan (QASC), was successfully synthesized and characterized. In comparison to conventional inhibitors, QASC demonstrated significantly enhanced performance in terms of suppressing bentonite hydration swelling and improving shale cuttings recovery efficiency.
2. Based on comprehensive microstructural analysis, the inhibition mechanism of QASC was systematically proposed. QASC can strongly adsorb onto negatively charged clay surfaces via synergistic electrostatic attraction and hydrogen bonding interactions, effectively reducing the zeta potential of sodium bentonite particles and suppressing diffuse double-layer formation. Furthermore, QASC molecules intercalate into bentonite interlayer galleries while simultaneously adsorbing onto basal surfaces, thereby creating a dual protective barrier against water invasion within the clay structure.
3. Although the synthesis of QASC shale inhibitor is costly (due to complex purification and quaternization processes for chitosan) and its inhibition efficacy tends to diminish in high-salinity environments, with long-term thermal stability requiring further improvement, it still demonstrates promising application prospects as a high-performance, environmentally friendly shale inhibitor for water-based drilling fluids.

## Figures and Tables

**Table 1:** Original drilling fluid formulation.

No.	Bentonite	FA367	BTM-2	AP-1	DSP-1	NP-1	Emulsified Asphalt	Barite
Content (wt%)	4	0.15	0.5%	0.5	1.5%	10.4	3	85

**Table 2:** Mass fraction of different elements.

Sample	Mass Fraction of Different Elements	
	C (%)	N (%)
CS	41.52	7.80
QASC	36.42	7.16

**Table 3:** The effects of QASC on drilling fluids.

Experimental Conditions	Formulation	AV (mPa·s)	PV (mPa·s)	YP (Pa)	YP/PV (Pa)	G_10s/10min_ (Pa/Pa)	FL_API_ (mL)
indoor temperature	Original formulation	80	59	19	0.3	1.5/5.0	2.6
	Original formulation +1% QASC	88	67	21	0.2	2.5/6.0	3.4
hot-rolled at 150 °C for 16 h	Original formulation	64	48	16	0.3	1.5/4.5	3.8
	Original formulation +1% QASC	80	63	17	0.3	2.0/5.0	4.4

## Data Availability

All data generated or analysed during this study are included in this article. No external datasets are required.
